# Supplementary material for: Immunogenic epitope scanning in bacteriolytic enzymes Pal and Cpl-1 and engineering Pal to escape antibody responses
Source: Front Immunol. 2023 Sep 15;14:1075774. doi: 10.3389/fimmu.2023.1075774 (PMC10540205; doi:10.3389/fimmu.2023.1075774)
Supplement: Supplementary file 1 [file DataSheet_1.docx]

**Supplementary Data**

**Supplementary Table S1.** **P-values for relative signals between the input sample v. tested group.** Only adj. p-values < 0.05 are presented. Adj. p-values between relative signal (ratio) of a measured oligopeptide after immunoprecipitation and before (control level in input sample), values were calculated by GraphPad Prism 9 using the Kruskal-Wallis test, adjusted for multiple comparisons (Benjamini, Krieger and Yekutieli). The control group is the library before immunoprecipitation.

| location of a tested oligopeptide in Cpl-1 or Pal [position of the first amino acid] | p-value for relative signals between input sample v. tested group | |
| --- | --- | --- |
|  | mouse | human |
|  |  |  |
| **Cpl-1** | | |
| 11 | <0.0001 | 0.0231 |
| 21 | 0.0023 | 0.0348 |
| 31 | 0.0018 | 0.0015 |
| 181 | 0.0116 | 0.0330 |
| 191 | 0.0011 | 0.0008 |
| 281 | <0.0001 | <0.0001 |
| 291 | 0.2164 | 0.0005 |
| 301 | <0.0001 | 0.0003 |
| **Pal** | | |
| 141 | <0.0001 | <0.0001 |
| 231 | 0.0011 | 0.0259 |
| 251 | 0.0001 | 0.0252 |

**Supplementary Table S2**. **Statistical analysis of Pal and Cpl-1 variants’ cross-reactivity.** Unpaired *t*-test between reactivity of specific IgG in shown groups, p-value adjusted using the Benjamini & Hochberg method, protocol proposed by Benjamini & Hochberg (1995) (Figure 2), 6 biological replicates in each group.

| Cpl-1 WT v. Cpl-1 v4 | p=0.0010 |
| --- | --- |
| Cpl-1 WT v. Cpl-1 v5 | p=0.00036 |
| Cpl-1 WT v. Cpl-1 v1 | p=0.016 |
| Cpl-1 WT v. Cpl-1 v2 | p=0.016 |
| Cpl-1 WT v. Cpl-1 v3 | p=0.0051 |
| Pal WT v. Pal v1 | p=0.0000024 |
| Pal WT v. Pal v3 | p=0.00000011 |
| Pal WT v. Pal v9 | p=0.00000018 |


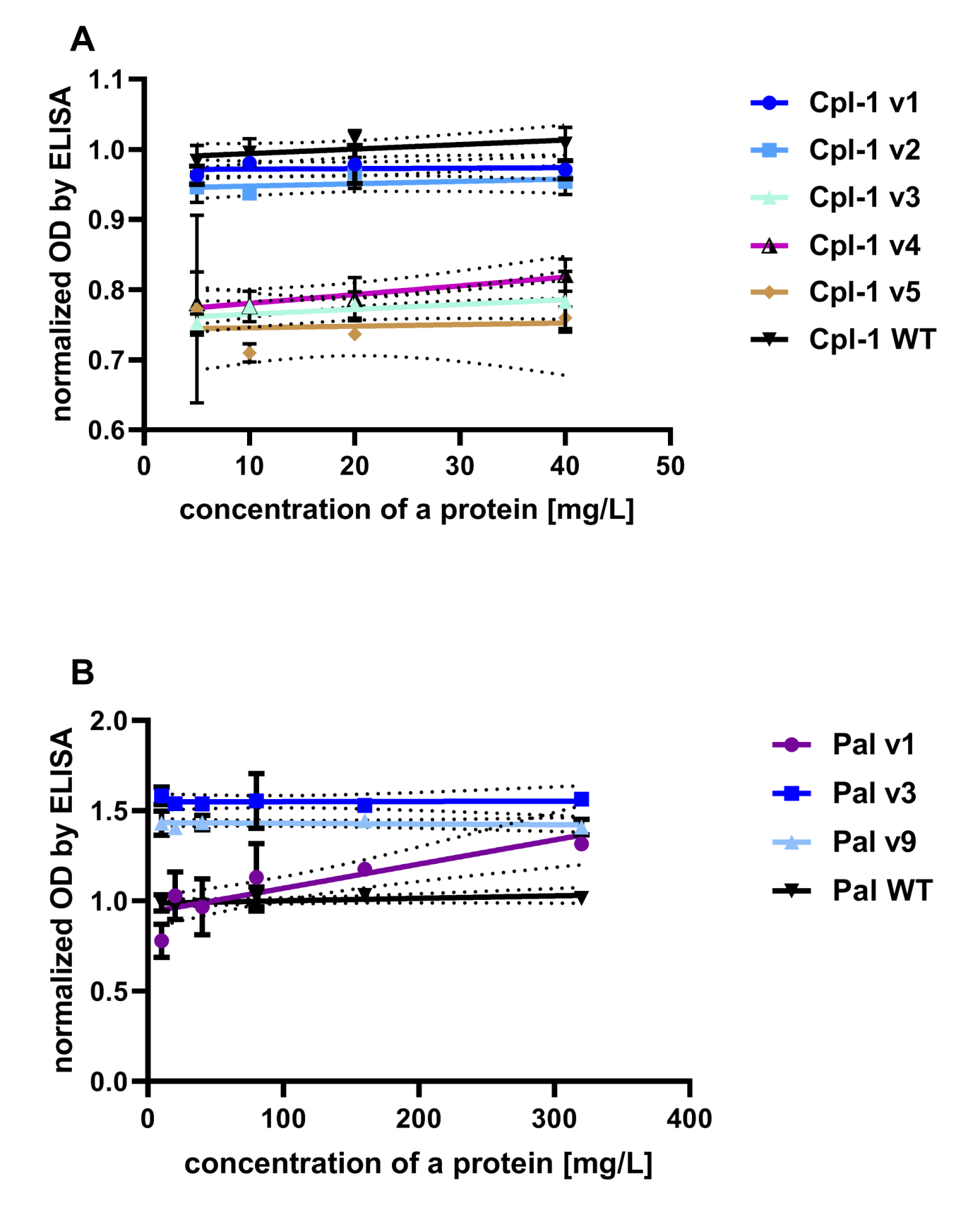


**Supplementary** **Figure S1. Levels of specific IgG in detected in wells coated with varying concentration of proteins.** The efficacy of well coating with our proteins had no significant effect on ELISA signal, thus significance of comparisons between ELISA signals could be assessed. **(A**) Normalized levels of specific IgG detected in wells coated with differing concentrations of Cpl-1 variants. (**B**) Normalized levels of specific IgG detected in wells coated with differing concentrations of Pal variants. OD readings by ELISA are normalized to the average signal from WT protein set to 1.0. Points and whiskers represent mean and standard deviation of 6 biological replicates. Line represent linear regression model (dotted line represent 95% confidence interval) calculated and plotted by GraphPad Prism 9.


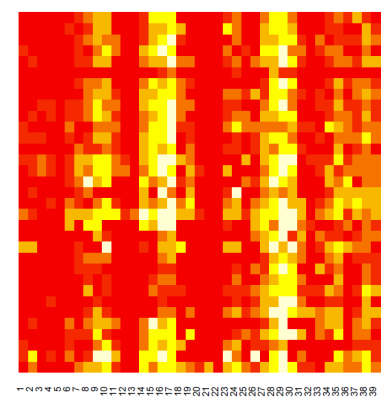


**Supplementary Figure S2**. **An examplary heatmap – graphical representation of data used for defining key amino acids within immunogenic regions (as demonstrated in Figure 3).** Here for Cpl-1 – oligopeptide no. 31 starting after the 300^th^ amino acid position in the protein. Each row represent one murine serum sample (33 mice in total), each column represent oligopeptide no 31 with a specific (number on x-axis) amino acid substituted with alanine (or glycine if it previously was an alanine). Colors show the ratio of detected signal between alanine-substituted and unsubstituted oligopeptides (mean of 6 biological replicates). White square means a ratio below 20%, red square show a ratio over 90% (no signal lost by substitution).

>Pal v1

MGVDIEKGVAWMQARKGRVSYSMDFRDGPDSYDCSSSMYYALRSAGASSAGWAVNTEYMHAWLIENGYELISENAPWDAKRGDIFIWGRKGASAGAGGHTGMFIDSDNIIHCNYAYDGISVNDHDERWYYAGQPYYYVYRLTNANAQPAEKKLGWQKDATGFWYARANGTYPKDEFEYIEENKSWFYFDDQGYMLAEKWLKHTDGNWYWFDRDGYMATSWKRIGESWYYFNRDGSMVTGWIKYYDNWYYCDATNGD**TFK**NAFIRYNDGWYLLLPDGRLADKPQFTVEPDGLITAKV

>Pal v2

MGVDIEKGVAWMQARKGRVSYSMDFRDGPDSYDCSSSMYYALRSAGASSAGWAVNTEYMHAWLIENGYELISENAPWDAKRGDIFIWGRKGASAGAGGHTGMFIDSDNIIHCNYAYDGISVNDHDERWYYAGQPYYYVYRLTNANAQPAEKKLGWQKDATGFWYARANGTYPKDEFEYIEENKSWFYFDDQGYMLAEKWLKHTDGNWYWFDRDGYMATSWKRIGESWYYFNRDGSMVTGWIKYYDNWYYCDATNGDMKSNAFIR**DKGL**WYLLLPDGRLADKPQFTVEPDGLITAKV

>Pal v3

MGVDIEKGVAWMQARKGRVSYSMDFRDGPDSYDCSSSMYYALRSAGASSAGWAVNTEYMHAWLIENGYELISENAPWDAKRGDIFIWGRKGASAGAGGHTGMFIDSDNIIHCNYAYDGISVNDHDERWYYAGQPYYYVYRLTNANAQPAEKKLGWQKDATGFWYARANGTYPKDEFEYIEENKSWFYFDDQGYMLAEKWLKHTDGNWYWFDRDGYMATSWKRIGESWYYFNRDGSMVTGWIKYYDNWYYCDATNGDMKSNAFIRYNDGWYLLLPDGRLA**GGA**QFTVEPDGLITAKV

>Pal v4

MGVDIEKGVAWMQARKGRVSYSMDFRDGPDSYDCSSSMYYALRSAGASSAGWAVNTEYMHAWLIENGYELISENAPWDAKRGDIFIWGRKGASAGAGGHTGMFIDSDNIIHCNYAYDGISVNDHDERWYYAGQPYYYVYRLTNANAQPAEKKLGWQKDATGFWYARANGTYPKDEFEYIEENKSWFYFDDQGYMLAEKWLKHTDGNWYWFDRDGYMATSWKRIGESWYYFNRDGSMVTGWIKYYDNWYYCDATNGDMKSNAFIRYNDGWYLLLPDGRLADKP**GLA**VEPDGLITAKV

>Pal v5

MGVDIEKGVAWMQARKGRVSYSMDFRDGPDSYDCSSSMYYALRSAGASSAGWAVNTEYMHAWLIENGYELISENAPWDAKRGDIFIWGRKGASAGAGGHTGMFIDSDNIIHCNYAYDGISVNDHDERWYYAGQPYYYVYRLTNANAQPAEKKLGWQKDATGFWYARANGTYPKDEFEYIEENKSWFYFDDQGYMLAEKWLKHTDGNWYWFDRDGYMATSWKRIGESWYYFNRDGSMVTGWIKYYDNWYYCDATNGD**AAA**NAFIR**AAAA**WYLLLPDGRLA**AAAAAA**VEPDGLITAKV

>Pal v6

MGVDIEKGVAWMQARKGRVSYSMDFRDGPDSYDCSSSMYYALRSAGASSAGWAVNTEYMHAWLIENGYELISENAPWDAKRGDIFIWGRKGASAGAGGHTGMFIDSDNIIHCNYAYDGISVNDHDERWYYAGQPYYYVYRLTNANAQPAEKKLGWQKDATGFWYARANGTYPKDEFEYIEENKSWFYFDDQGYMLAEKWLKHTDGNWYWFDRDGYMATSWKRIGESWYYFNRDGSMVTGWIKYYDNWYYCDATNGD**TFK**NAFIR**DKGL**WYLLLPDGRLA**GGAGLA**VEPDGLITAKV

>Pal v7

MGVDIEKGVAWMQARKGRVSYSMDFRDGPDSYDCSSSMYYALRSAGASSAGWAVNTEYMHAWLIENGYELISENAPWDAKRGDIFIWGRKGASAGAGGHTGMFIDSDNIIHCNYAYDGISVNDHDERWYYAGQPYYYVYRLTNANAQPAEKKLGWQKDATGFWYARANGTYPKDEFEYIEENKSWFYFDDQGYMLAEKWLKHTDGNWYWFDRDGYMATSWKRIGESWYYFNRDGSMVTGWIKYYDNWYYCDATNGD**TFK**NAFIRYNDGWYLLLPDGRLA**GGA**QFTVEPDGLITAKV

>Pal v8

MGVDIEKGVAWMQARKGRVSYSMDFRDGPDSYDCSSSMYYALRSAGASSAGWAVNTEYMHAWLIENGYELISENAPWDAKRGDIFIWGRKGASAGAGGHTGMFIDSDNIIHCNYAYDGISVNDHDERWYYAGQPYYYVYRLTNANAQPAEKKLGWQKDATGFWYARANGTYPKDEFEYIEENKSWFYFDDQGYMLAEKWLKHTDGNWYWFDRDGYMATSWKRIGESWYYFNRDGSMVTGWIKYYDNWYYCDATNGDMKSNAFIR**LAGL**WYLLLPDGRLADKPQFTVEPDGLITAKV

>Pal v9

MGVDIEKGVAWMQARKGRVSYSMDFRDGPDSYDCSSSMYYALRSAGASSAGWAVNTEYMHAWLIENGYELISENAPWDAKRGDIFIWGRKGASAGAGGHTGMFIDSDNIIHCNYAYDGISVNDHDERWYYAGQPYYYVYRLTNANAQPAEKKLGWQKDATGFWYARANGTYPKDEFEYIEENKSWFYFDDQGYMLAEKWLKHTDGNWYWFDRDGYMATSWKRIGESWYYFNRDGSMVTGWIKYYDNWYYCDATNGD**TFG**NAFIRYNDGWYLLLPDGRLADKPQFTVEPDGLITAKV

>Cpl-1 v1

MVKKNDLFVDVSSHNGYDITGILEQMGTTNTIIKISESTTYLNPCLSAQVEQSNPIGFYHFARFGGDVAEAEREAQFFLDNVPMQVKYLVLDYEDDPSGDAQANTNACLRFMQMIADAGYKPIYYSYKPFTHDNVDYQQILAQFPNSLWIAGYGLNDGTANFEYFPSMDGIRWWQYSSNPFDKNIVLLDDEEDDKPKTAGTWKQDSKGWWFRRNNGSFPYNKWEKIGGVWYYFDSKGYCLTSEWLKDNEKWYYLKDNGAMATGWVLVGSEWYYMDDSGAMVTGWVKYKNNWYYMTNERGNMVSNEFIK**DAW**GWYFMNTNGELADNPSFTKEPDGLITVA

>Cpl-1 v2

MVKKNDLFVDVSSHNGYDITGILEQMGTTNTIIKISESTTYLNPCLSAQVEQSNPIGFYHFARFGGDVAEAEREAQFFLDNVPMQVKYLVLDYEDDPSGDAQANTNACLRFMQMIADAGYKPIYYSYKPFTHDNVDYQQILAQFPNSLWIAGYGLNDGTANFEYFPSMDGIRWWQYSSNPFDKNIVLLDDEEDDKPKTAGTWKQDSKGWWFRRNNGSFPYNKWEKIGGVWYYFDSKGYCLTSEWLKDNEKWYYLKDNGAMATGWVLVGSEWYYMDDSGAMVTGWVKYKNNWYYMTNERGNMVSNEFIKSGKGWYF**R**NTNGELADNPSFTKEPDGLITVA

>Cpl-1 v3

MVKKNDLFVDVSSHNGYDITGILEQMGTTNTIIKISESTTYLNPCLSAQVEQSNPIGFYHFARFGGDVAEAEREAQFFLDNVPMQVKYLVLDYEDDPSGDAQANTNACLRFMQMIADAGYKPIYYSYKPFTHDNVDYQQILAQFPNSLWIAGYGLNDGTANFEYFPSMDGIRWWQYSSNPFDKNIVLLDDEEDDKPKTAGTWKQDSKGWWFRRNNGSFPYNKWEKIGGVWYYFDSKGYCLTSEWLKDNEKWYYLKDNGAMATGWVLVGSEWYYMDDSGAMVTGWVKYKNNWYYMTNERGNMVSNEFIKSGKGWYFMNTNGELADNPSF**LQP**PDGLITVA

>Cpl-1 v4

MVKKNDLFVDVSSHNGYDITGILEQMGTTNTIIKISESTTYLNPCLSAQVEQSNPIGFYHFARFGGDVAEAEREAQFFLDNVPMQVKYLVLDYEDDPSGDAQANTNACLRFMQMIADAGYKPIYYSYKPFTHDNVDYQQILAQFPNSLWIAGYGLNDGTANFEYFPSMDGIRWWQYSSNPFDKNIVLLDDEEDDKPKTAGTWKQDSKGWWFRRNNGSFPYNKWEKIGGVWYYFDSKGYCLTSEWLKDNEKWYYLKDNGAMATGWVLVGSEWYYMDDSGAMVTGWVKYKNNWYYMTNERGNMVSNEFIK**DAW**GWYF**R**FTNGELADNPSF**LQP**PDGLITVA

>Cpl-1 v5

MVKKNDLFVDVSSHNGYDITGILEQMGTTNTIIKISESTTYLNPCLSAQVEQSNPIGFYHFARFGGDVAEAEREAQFFLDNVPMQVKYLVLDYEDDPSGDAQANTNACLRFMQMIADAGYKPIYYSYKPFTHDNVDYQQILAQFPNSLWIAGYGLNDGTANFEYFPSMDGIRWWQYSSNPFDKNIVLLDDEEDDKPKTAGTWKQDSKGWWFRRNNGSFPYNKWEKIGGVWYYFDSKGYCLTSEWLKDNEKWYYLKDNGAMATGWVLVGSEWYYMDDSGAMVTGWVKYKNNWYYMTNERGNMVSNEFIK

**Supplementary Figure S3. Full list of variants with modified epitopes identified by EndoScan in fasta format.**


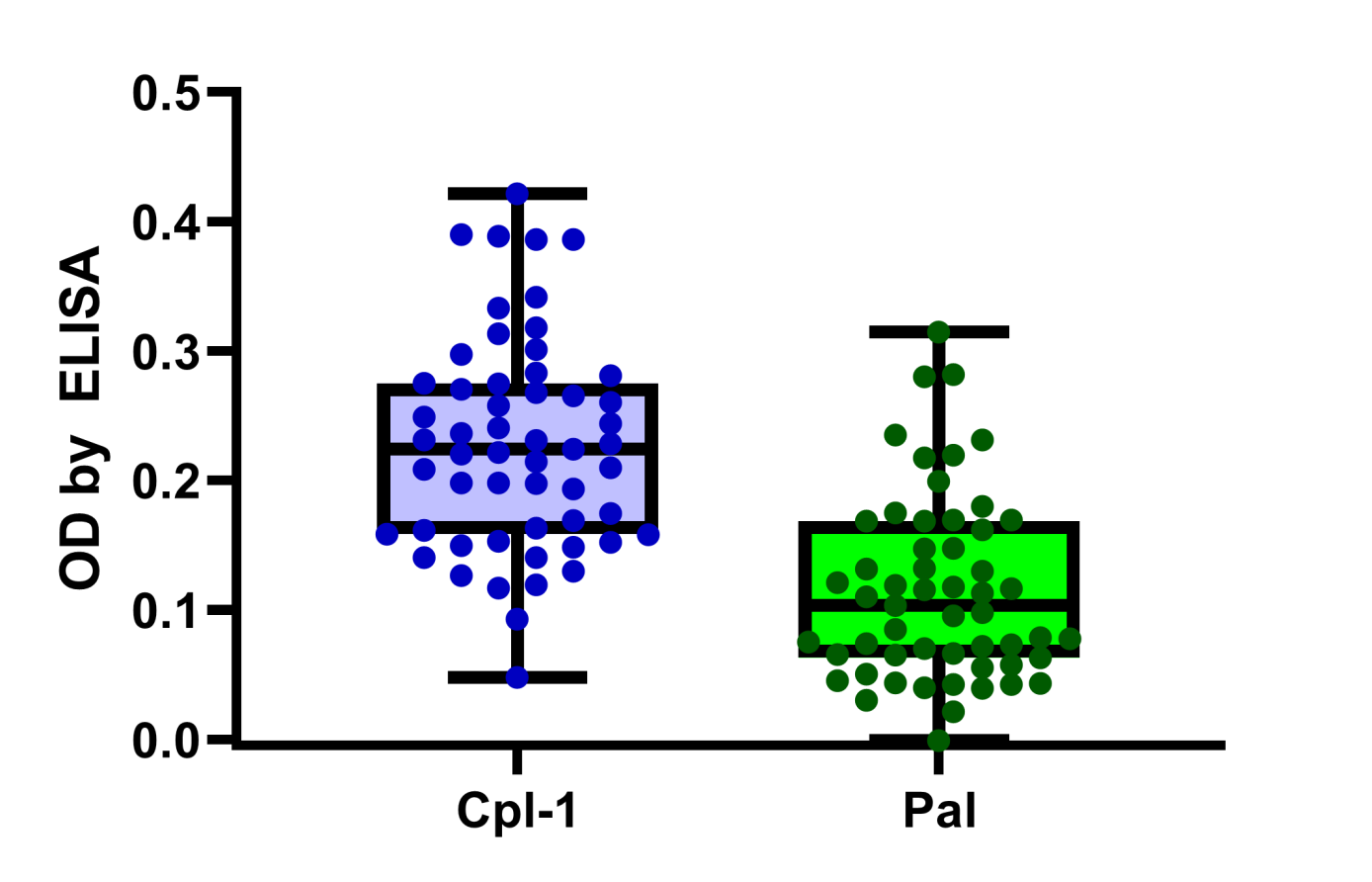


**Supplementary Figure S4.** **Distribution of endolysin-specific antibodies in humans, european population** (N=56); dots represent values of individual samples (mean of 3 technical replicates); line and whiskers represent median and min or max read respectively. Upper and lower border of box represent 75^th^ and 25^th^ quantile of data. One serum sample showed an abnormally high reading against both proteins (>0.6), resulting in this sample being identified as an outlier (ROUT method, GraphPad Prism 9, cut-off - 1%) and it’s not plotted.
